# Association Study between BDNF Gene Polymorphisms and Autism by Three-Dimensional Gel-Based Microarray

**DOI:** 10.3390/ijms10062487

**Published:** 2009-06-02

**Authors:** Lu Cheng, Qinyu Ge, Pengfeng Xiao, Beili Sun, Xiaoyan Ke, Yunfei Bai, Zuhong Lu

**Affiliations:** 1 State Key Laboratory of Bioelectronics, Southeast University, Nanjing, 210096, China; E-Mails: chenglu@seu.edu.cn (L.C.); xiaopf@seu.edu.cn (P.X.); sunbl@seu.edu.cn (B.S.); whitecf@seu.edu.cn (Y.B.); 2 Key Laboratory of Child Development and Learning Science, Ministry of Education, Southeast University, Nanjing, 210096, China; E-Mail: geqinyu@seu.edu.cn (Q.G.); 3 Child Mental Health Research Center of Nanjing Brain Hospital affiliated of Nanjing Medical University, Nanjing, 210029, China; E-Mail: ke_xiaoyan@yahoo.com.cn (X.K.)

**Keywords:** DNA microarray, single nucleotide polymorphism (SNP), autism, brain-derived neurotrophic factor (BDNF)

## Abstract

Single nucleotide polymorphisms (SNPs) are important markers which can be used in association studies searching for susceptible genes of complex diseases. High-throughput methods are needed for SNP genotyping in a large number of samples. In this study, we applied polyacrylamide gel-based microarray combined with dual-color hybridization for association study of four BDNF polymorphisms with autism. All the SNPs in both patients and controls could be analyzed quickly and correctly. Among four SNPs, only C270T polymorphism showed significant differences in the frequency of the allele (χ^2^ = 7.809, *p =* 0.005) and genotype (χ^2^ = 7.800, *p =* 0.020). In the haplotype association analysis, there was significant difference in global haplotype distribution between the groups (χ^2^ = 28.19, *p = 3.44e-005*). We suggest that BDNF has a possible role in the pathogenesis of autism. The study also show that the polyacrylamide gel-based microarray combined with dual-color hybridization is a rapid, simple and high-throughput method for SNPs genotyping, and can be used for association study of susceptible gene with disorders in large samples.

## 1. Introduction

Single nucleotide polymorphisms (SNPs) are the most frequently occurring genetic variation in the human genome, with the total number reported in public SNP databases currently exceeding nine million [1,2]. SNPs are important markers which can account for phenotypic diversity, influencing risk of certain disease, and variable response to drugs and the environment. Genetic association studies utilizing SNP markers are expected to allow identification of genetic factors responsible for complex diseases that will facilitate early diagnosis, prevention, prognosis and, possibly, treatment of human diseases [3,4]. Due to the large number of SNPs, methods that allow high-throughput, cost-effective and fast detection are needed. A variety of techniques have been developed to type SNPs in a high-throughput fashion [5–7], DNA microarray is one of the high-throughput platform for SNPs analysis.

DNA microarrays allow us to acquire abundant information at once. A significant advantage of performing assays in microarray formats is that the costs of genotyping are reduced, because many SNPs are analyzed simultaneously, and the reaction volume employed on the microarrays is small. In general, there are two microarray-based methods for SNPs genotyping. One is arraying thousands of short oligonucleotides to substrates for detection of thousands upon thousands of possible SNPs in target DNA [8–10]. This method is suited for genotyping a large amount of markers in a limited number of individuals. In many cases, association analysis needs several markers of interest genes for large samples, so the first kind of microarray is not adapted to it. The other kind of microarray approach involves arraying samples DNA including amplified PCR products to substrates to detect SNPs in a large number of samples [11]. Our lab has successfully developed 3-dimentional (3-D) polyacrylamide gel-based microarray hybridized with dual-color fluorescent probes to genotype several SNPs in hundreds and thousands of samples, which belongs to the second kind microarray for SNP genotyping [12,13].

Many genetic variants and environmental exposures can influence the occurrence and progression of psychiatric disorders, including autism [14]. Autism is a permanent developmental disorder characterized by marked deficits in communication and social interaction skills, language impairment, and abnormal behavior. After decades of research, the cause of autism is still unknown. Several studies have provided evidence suggesting that autism is a pathophysiological process arising from the interaction of an early environmental insult with a genetic predisposition for the disease [15]. Family and twin studies have produced the evidence for a higher genetic risk for autism in siblings of autistic probands than in the general population, providing additional support for the hypothesis of the importance of genetic risk factors in autism [16]. Although there has been a significant increase in autism genetics research recently, validated susceptibility genes for autism have yet to be identified. The identification of autism-susceptibility genes will not only assist in the identification and/or development of better medications that can help improve the health and neurodevelopment of children with autism, but will also allow for better perinatal diagnosis.

Brain-derived neurotrophic factor (BDNF), a small dimeric protein and a member of the neurotrophic factor family, is expressed widely throughout the mammalian brain [17]. BDNF is involved in the survival and differentiation of dopaminergic neurons in the developing brain, and plays an important role in the formation and plasticity of synaptic connections [18]. BDNF is also trophic for serotonergic neurons, and abnormalities in serotonin levels are the most common biochemical findings in autism [19]. In this study, we examined the SNP- and haplotypic-association of BDNF gene with autism in case-control study. Four SNPs (rs988748, rs2049046, C270T and rs6265) were selected for association study.

## 2. Results and Discussion

### 2.1. Gel-based microarray for genotyping

**Figure 1.**
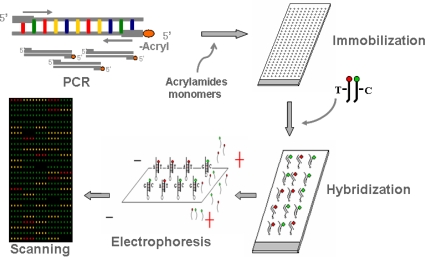
Schematic outline of gel-immobilization microarray approach for SNP genotyping in a large number of samples. The platform for SNP genotyping mainly involves five steps: polymerase chain reaction (PCR), immobilization of PCR products, hybridization, electrophoresis of microarray and scanning for genotyping.

Four SNPs (rs988748, rs2049046, C270T and rs6265) of BDNF gene were selected for our association study. Genotyping was acquired by three-dimentional (3-D) polyacrylamide gel-based microarray hybridized with dual-color fluorescent probes. Schematic outline of gel-immobilization microarray approach for high-throughput genotyping is shown in Figure 1. The whole SNP detection system mainly involves five steps: polymerase chain reaction (PCR), immobilization of PCR products, hybridization, electrophoresis of microarray and scanning for genotyping.

**Figure 2.**
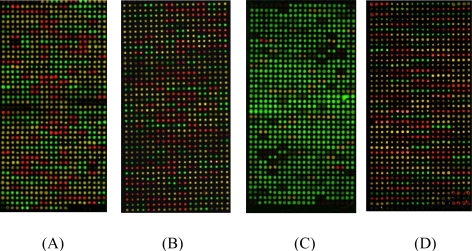
Hybridized image of four SNPs of BDNF gene for part of samples. The images acquired by the confocal scanner which was fitted with filters for Cy3 and Cy5. The green spots indicate wild homozygous, the red spots indicate mutant homozygous, and the yellow spots indicate heterozygote. (A) scan image of rs988748, (B) scan image of rs2049046, (C) scan image of C270T, (D) scan image of rs6265.

After overlapping Cy3 and Cy5 image, a variety of different fluorescent signals of SNPs loci should be shown for three genotypes. The homozygous wild type yielded a strongly fluorescing Cy3 spots (green fluorescence). The homozygous mutant type yielded a strongly fluorescing Cy5 spots (red fluorescence). As the heterozygote yielded both fluorescing Cy3 and Cy5 spots, a strongly ‘yellow’ fluorescence was shown. The scan images of the four SNPs for part of samples are shown in Figure 2.

Our lab has successfully developed dual-color hybridization based microarray to genotype several SNPs in hundreds and thousands of samples. The method was based on immobilizing amino modified PCR products onto a poly-l-lysine coated glass slides to fabricate a microarray, which was then interrogated by hybridization with dual-color probes to determine the SNP genotype of each sample [11]. Three genotypes could be analyzed easily according to green, red and yellow colors. It was confirmed that dual-color fluorescence hybridization was an accurate method for SNPs genotyping. Because of the low immobilization efficiency of nucleic acids in two-dimensional (2-D) substrates, purification and concentration of PCR products by commercial purification cartridges was required to achieve high quantity in microarray spotting. Generally 200 μL of PCR products was needed, which were concentrated to 20 μL for genotyping. The purification steps increase not only the cost and labor needed, but also the risk of introducing contamination, especially in the process of purifying large amounts of PCR products for a large number of samples. In order to overcome the limitations of the planar microarray format, polyacrylamide gel-based microarray technology was developed [12]. As the polyacrylamide gel-based microarray has long branching capillaries that can bind DNA molecules on to solid 3-D structure substrate, this substrate can provide surfaces with very high probe density (200 fmol/mm^2^) [20], which is several hundred fold larger than that of 2-D substrate. Only 20 μL PCR reaction volume is needed for genotyping, and concentration and purification of PCR products can be abandoned. On the other hand, the gel-based microarray is porous, which can offer liquid-liquid environment for hybridization like in solution, so the efficiency of hybridization is enhanced much more than that of 2-D microarray.

Acrylamide-modified nucleic acids polymerized with acrylamide monomers to fabricate gel-based microarray was first described by Rehman *et al*. [20]. The gel-based microarray has many advantages, but the method has not been accepted and used broadly for many years. The main problem of this method is that it was unable to control the polymerization process, so that the viscosity of the prepolymer solution and the concentration of the probes will change during the spotting, as the polymerization of prepolymer was initiated immediately after mixing. Thus it was difficult to spot the mixed prepolymers homogeneously onto the slide to acquire a large scale microarray. According to the polymerization mechanism, the prepolymer does not polymerize immediately at room temperature without TEMED, so we modified Rehman’s method by controlling the polymerization to prepare the sample microarray. Excluding TEMED, acrylamide modified PCR products were mixed with acrylamide monomers, glycerol, 1XTBE, APS into prepolymer mixture. The prepolymer mixture was chemically stable, both the concentration of the nucleic acid and the viscosity of the solution remained unchanged for a long time. Thus, the mixture could be spotted stably in a period long enough for forming homogeneous microarray spots. After spotting, the prepared slides were placed in a vacuum chamber with TEMED under reduced pressure, so that TEMED was vaporized and diffused into the gel spots to induce polymerization. With this improvement, the polymerization process could be controlled, and a high quality gel-based DNA microarray could be fabricated.

Though many advantages had the polyacrylamide gel-based microarray, there were still some drawbacks for wide application, especially porous structure intensively adsorbed the labeled nucleic acid, which could lead to high backgrounds. Removing the adsorbed nucleotide and the other nonspecific bindings by the conventional washing steps was not easy, especially for removing single base mismatched probes. In our method, electrophoresis of the slides was used instead of the conventional washing procedure in the post-hybridization process [12]. During electrophoresis, the hybrids (the dual-spiral structures formed in hybridization) would be unchained when probes were continuously removed. In this case, not only the non-specific binding probes, but also the probes participating in hybridization would be removed. The stability of the perfectly matched hybrid is higher than that of the mismatched one, so the fluorescence signal change of the hybrid with mismatched target is faster than that of matched one. Under optimal conditions, such as the optimized electrophoresis temperature, voltage and current, only the perfectly matched sequences had the distinct hybridization signals.

### 2.2. Association study

A total of 124 autism patients and 120 matched controls were involved in the association study. The age and sex distributions for the autism and control populations were similar (*p* > 0.05). The genotypes and the allele distributions and Hardy – Weinberg equilibrium tests for the four BDNF polymorphisms in the autism patients and controls are presented in Table 1. No polymorphism showed evidence of deviation from the expected values at the Hardy – Weinberg equilibrium test for either cases or controls (*p* > 0.05). Among the four BDNF polymorphisms, only C270T showed significant differences in the frequency of the allele (*p =* 0.005) and genotype (*p =* 0.020) between autism patients and healthy controls (Table 1). The four markers were found to be in strong LD to each other both in the patient group (D’ > 0.7) and the control group (D’ > 0.7). The results of global case-control haplotype analysis and comparisons of individual haplotypes between groups are presented in Table 2. Haplotype was organized from 5′ to 3′: rs988748, rs2049046, C270T, rs6265. Only haplotypes with frequency > 0.03% were considered in analysis. Global case-control haplotype analysis showed that there was significant difference in haplotype distribution (χ^2^ = 28.19, *p = 3.44e-005*). Individual haplotype analysis showed that the frequencies of three haplotypes (CTTG, CACA and GTCG) had significant difference between groups (*p* < 0.05).

In the brain, BDNF has been implicated in development, neural regeneration, synaptic transmission, synaptic plasticity and neurogenesis [21,22]. Elevated BDNF expression has been observed in the brain [23], blood [24] and serum [25,26] of autistic individuals, compared to healthy controls. Another line of evidence concerned that BDNF had a possible role in the pathogenesis of autism through its neurotrophic effects on the serotonergic system. The serotonergic system has been found to be developmentally dysregulated in autism, and BDNF has been confirmed to play a critical role in the serotonergic function [27,28]. BDNF has been implicated having correlation with some psychiatric disorders, including depression [29], obsessive compulsive disorder [30], attention deficit hyperactivity disorder (ADHD) [31,32], and anxiety-related personality traits [33]. In the present study, we investigated whether a relationship existed between genetic variants of BDNF gene and autism.

The human BDNF gene is located on the short arm of chromosome 11 at the boundary of 11p13 and 11p14 [34]. Four SNPs within the BDNF gene were selected for association study, which had been confirmed to be associated with other psychiatric disorders, such as schizophrenia, Alzheimer’s disease, ADHD and so on. We would like to know whether the four SNPs had any relationship with autism too. Among the four SNPs, only C270T showed significant differences in the frequency of the allele and genotype between patients and controls. Increased transmission of the T allele of C270T polymorphism was found in autism samples (autism, 12.1%, control, 5.0%). The C270T polymorphism is located in the 5′-noncoding region of intron 1 and maybe influences changes in BDNF expression [35]. Further studies are needed to determine whether the T allele is correlated with elevated BDNF expression in autistic patients.

There was no significant difference of rs988748, rs2049046 and rs6265 polymorphisms between the patients and controls. The rs988748 and rs2049046 polymorphisms are located in the promoter region. The SNP rs6265 has been identified at nucleotide 196 within the 5′ precusor peptide (proBDNF) sequence that causes an amino acid substitution of valine to methionine at codon 66 (Val66Met) [36]. The rs6265 polymorphism has been reported to be associated with obsessive compulsive disorder [30], attention deficit hyperactivity disorder (ADHD) [31,32], anxiety-related personality traits [33] and other childhood-onset mood disorder [37]. This SNP has also been suggested to have a role in the hippocampal and prefrontal cortex functions involved in human memory and learning [38,39]. However, we did not observe significant association of rs6265 polymorphism with autism. It is suggested that further study is needed in a larger sample.

We also found significant difference of global haplotype and three individual haplotype in case-control study. Increased transmission of the haplotypes CTTG, CACA, GTCG were showed in the autism group. As these haplotypes, especially CACA (4.1%) and GTCG (3.5%) were also rare in the patients group, it was hard to confirm that the increased transmission of the haplotypes were associated with autism. A similar study by Nishimura had been done about two years ago. Twenty-five SNPs were selected for analysis in trio families of the Caucasian population. They found some haplotype blocks show significant associations in the autism, but didn’t find significant associations from any single SNP [19]. Our findings were based on case-control study in Chinese population. Three SNPs were same to Nishimura’s study, except C270T polymorphism. Yet only C270T show significant association in the autism.

If BDNF is an uncommon disease locus or one with a small effect, our power to detect a gene would be reduced. A further study in a larger sample is needed to fully resolve the possible involvement of the genetic variants of the BDNF gene in autism.

## 3. Experimental Section

### 3.1. Subjects

In the present study, a total of 124 Chinese Han autism patients were recruited at Child Mental Health Research Center of Nanjing Brain Hospital, Nanjing Medical University, P.R. China. The diagnosis of the autistic disorder was based on the criteria of the Fourth Edition of Diagnostic and Statistical Manual of Mental Disorders (DSM-IV), the Autism Diagnostic Inventory-Revised (ADI-R), and the Childhood Autism Rating Scale (CARS). In these patients, 107 (86.3%) were male and 17 (13.7%) were female, and the mean age was 5.36 and 4.57 years. A group of 120 control subjects matched age and sex were recruited from community. None of control subjects had a history of diagnosed neurological disorder or genetic, major medical condition. All participants signed a written informed consent and all research procedures were approved by the Ethics Committee of Nanjing Brain Hospital.

### 3.2. Genotyping

The genomic DNA was extracted from the peripheral blood leukocytes using an improved KI salting-out procedure. Four SNPs (rs988748, rs2049046, C270T and rs6265) of BDNF gene were selected for association study. Genotyping was acquired by 3-dimentional (3-D) polyacrylamide gel-based microarray hybridized with dual-color fluorescent probes. Schematic outline of gel-immobilization microarray approach for high-throughput genotyping is shown in Figure 1. The whole SNP detection system mainly involves five steps: polymerase chain reaction (PCR), immobilization of PCR products, hybridization, electrophoresis of microarray and scanning for genotyping. The details of each step are explained as follows.

#### 3.2.1. PCR Amplification

PCR primers were designed using primer Premier 5.0 software based on published DNA sequence. The primers were synthesized and HPLC purified in TaKaRa Company (P.R. China). All reverse primers were modified with acrylamide group at 5′-terminal in order to covalently attach with the polyacrylamide gel. The sequences of all the primers and the predicted size of the PCR amplified fragments are shown in Table 3. Amplification was carried out in a final volume of 2 μL, consisting of 1.0 × PCR buffer (which contain 10 mM Tris-HCl, 50 mM KCl), 1.2 mM MgCl_2_, 200 μM dNTPs, 0.5 μM of each primer, 1 U Taq polymerase and 50 ng of genomic DNA. The profile consisted of an initial melting step of 4 min at 94 °C, followed by 35 cycles of 30 seconds at 94 °C, 30 seconds at 57 °C, and 30 seconds at 72 °C, with a final extension step of 5 min at 72 °C. After PCR amplification, PCR products were processed by ethanol precipitation.

#### 3.2.2. Immobilization of PCR products

Acryl-modified slides were prepared in advance. Glass slides were cleaned by soaking in 10% aqueous nitric acid for 2 h. Slides were then rinsed with water and acetone and air dried. The cleaned slides were soaked in 10% 3-methacryloxypropyltrimethoxy silane (Sigma Corporation, USA) in acetone for 1 h, and were washed in acetone and air dried for use [20].

Acrylamide-modified PCR products were dissolved with the solutions which contained 3% acrylamide monomer (29:1 acrylamide:bis-acrylamide), 30% glycerol, 1% APS, and then were spotted on the 3-methacryloxypropyltrimethoxy silane modified glass slides using a microarrayer (Captial Biochip Corporation, P.R. China). After spotting, the slide was placed into a humid sealed chamber in which a well containing TEMED was deposited in advance. The pressure in the sealed chamber was reduced to about 1,000 Pascal (Pa), and this pressure was maintained for 20 minutes at room temperature. Under this pressure, TEMED was vaporized and diffused into the spots and onto the slide surfaces to induce the copolymerization between acrylamide groups and acryl groups. Following the attachment, the slide was incubated in 0.1 M NaOH for electrophoresis eight min to obtain single stranded DNA (ssDNA) for hybridization and then incubated in 1XTBE for electrophoresis eight minutes for removing NaOH [12]. The slides were prepared for hybridization.

#### 3.2.3. Hybridization

**Figure 3.**
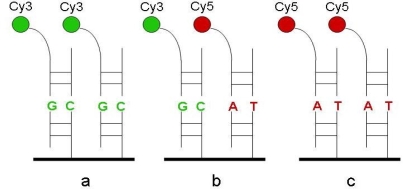
A schematic outline of SNP genotyping approach using dual-color fluorescence hybridization. (A) homozygous wild type; (B) heterozygote type; (C) homozygous mutant type.

For every SNP locus, a pair of probes were designed which could be matched with polymorphism part of the targets and labeled with the Cy3 or Cy5 respectively (Figure 3). The length of probes ranged from 13–15nt and the Tm value were adjusted within the range of 37–42 °C. The sequences of the probes were shown in Table 1. For every SNP genotyping, a pair of labeled probes were mixed in equimolar amounts and suspended in unihybridization solution (3:1 dilution) with the finial concentration of 2μM. Hybridization was performed in a moist chamber at 37 °C for 2–4 hours.

#### 3.2.4. Post-hybridization

After hybridization, the slides were subjected to electrophoresis (DYY-6C model electrophoresis cell, Beijing Liuyi Instrument Factory, P.R. China) under 2 V/cm for eight min in 1×TBE buffer at 4 ºC. After electrophoresis was completed, the slide was rinsed in water and air dried.

#### 3.2.5. Scanning and genotyping

The hybridization slides were scanned at 70% laser power and 65% PMT Gain with confocal scanner (Luxscan-10K/A, CapitalBio Company, P.R. China) after the above treatment. The scanner was fitted with filters for Cy3 and Cy5. Images were analyzed with QuantArray software.

### 3.3. Statistical analysis

Hardy Weinberg equilibrium (HWE), linkage disequilibrium (LD), haplotype construction and comparison in allele, genotype, and haplotype distribution between patients and controls were tested by SHEsis program (http://analysis.bio-x.cn/myAnalysis.php) [40]. The statistical significance was defined by p < 0.05. The option ‘drop rare haplotypes’ was used to restrict the analysis of haplotypes with a frequency < 3%.

## 4. Conclusions

It is concluded that the polyacrylamide gel-based microarray combined with dual-color hybridization is a rapid, simple and high-throughput method for SNPs genotyping, and has been successfully used for association study of BDNF gene with autism. The present study suggests that BDNF may play a role in the pathogenesis of autism through its neurotrophic effects on central nervous system. However, further studies including replication of these findings in a large sample, the association of polymorphisms with BDNF expression and the functional impact of BDNF in autism are warranted.

## Figures and Tables

**Table 1.** HWE test, genotype distribution and allele frequencies of the BDNF genetic polymorphisms for autism (n = 124) and controls (n = 120).

SNP	Group	HWE	Genotype (frequency %)			*p* value	Allele (frequency %)		*p* value	OR^a^	95%CI^b^
			CC	CG	GG		C	G			
rs988748	Autism	0.175	33(0.266)	69(0.556)	22(0.177)	0.228	135(0.544)	113(0.456)	0.807	1.045	0.732~1.492
	Control	0.293	37(0.308)	54(0.450)	29(0.242)		128(0.533)	112(0.467)			

			AA	AT	TT		A	T			
rs2049046	Autism	0.208	28(0.226)	69(0.556)	27(0.218)	0.191	125(0.504)	123(0.496)	0.710	0.935	0.655~1.334
	Control	0.207	36(0.300)	53(0.442)	31(0.258)		125(0.521)	115(0.479)			

			CC	CT	TT		C	T			
C270T	Autism	0.876	96(0.774)	26(0.210)	2(0.016)	***0.020***	218(0.879)	30(0.121)	***0.005***	0.382	0.191~0.766
	Control	0.564	108(0.900)	12(0.100)	0(0.000)		228(0.950)	12(0.050)			

			AA	AG	GG		A	G			
rs6265	Autism	0.129	22(0.177)	70(0.565)	32(0.258)	0.274	114(0.460)	134(0.540)	0.734	0.940	0.658~1.342
	Control	0.481	29(0.242)	56(0.467)	35(0.292)		114(0.475)	126(0.525)			

a: Odds Ratio.

b: Confidence Intervals.

**Table 2.** Inferred haplotype frequency in BDNF gene and haplotype comparison between autism patients and controls.

Haplotype	case	control	chi	*p* value	OR[95%CI]
G A C A	96.09(0.387)	107.30(0.447)	1.300	0.2543281	0.808 [0.560~1.166]
C A C G	9.21(0.037)	13.51(0.056)	0.901	0.342672	0.696 [0.293~1.650]
C T C G	88.96(0.359)	104.14(0.434)	2.296	0.129761	0.752 [0.520~1.088]
C T T G	21.99(0.089)	7.81(0.033)	7.056	***0.007923***	2.972 [1.286~6.868]
C A C A	10.26(0.041)	0.00(0.000)	10.382	***0.001281***	—
G T C G	8.58(0.035)	0.00(0.000)	8.767	***0.003082***	

Global result: Total control = 240.0, total case = 248.0, χ2 = 28.194, df = 5, *p* = 3.44e-005					

Haplotype is organized from 5′ to 3′: rs988748, rs2049046, C270T and rs6265. Only haplotypes with frequency > 0.03% are listed in this table.

**Table 3.** Primer sequence and probe sequence of four SNPs of BDNF gene.

SNP ID	Primer sequence (5′-3′)	Fragment size (bp)	Probe sequence
rs988748	F: 5′-TAGGGTTCCTCCAGTCCTTT	250	5′-Cy3-GGGTCT*C*TGGGGT
	R: ’-Acryl-CAGCACAGATGGCAGAGTTTA		5′-Cy5-GGGTCT*G*TGGGGT
rs2049046	F: 5′-CAGGAGGAGGGACCTTCATT	293	5′-Cy3-CCAGGG*A*CTCCAA
	R: 5′-Acryl-AGCCTTTCGGGTTCTCATTT		5′-Cy5-CCAGGG*T*CTCCAA
C270T	F: 5′-CAGAGGAGCCAGCCCGGTGCG	213	5′-Cy3-CTCCAC*C*TCCTGC
	R: 5′-Acryl-CTCCTGCACCAAGCCCCATTC		5′-Cy5-CTCCAC*T*TCCTGC
rs6265	F: 5′-AAACATCCGAGGACAAGGTG	246	5′-Cy3-GAACAC*A*TGATAG
	R: 5′-Acryl-AGAAGAGGAGGCTCCAAAGG		5′-Cy5-GAACAC*G*TGATAG
